# Effect of surface and porosity of biochar on water holding capacity aiming indirectly at preservation of the Amazon biome

**DOI:** 10.1038/s41598-018-28794-z

**Published:** 2018-07-16

**Authors:** Estela M. C. C. Batista, Juliana Shultz, Tassya T. S. Matos, Mayara R. Fornari, Thuany M. Ferreira, Bruno Szpoganicz, Rilton A. de Freitas, Antonio S. Mangrich

**Affiliations:** 1 Federal University of Paraná, Department of Chemistry, Curitiba, PR 81531-980 Brazil; 2 Federal University of Santa Catarina, Department of Chemistry, Florianópolis, SC 88040-900 Brazil; 3 National Institute of Science and Technology, Energy and Environment (INCT E&A), Salvador, BA 40170-115 Brazil

## Abstract

As part of efforts to reduce pressure on the Amazon and other biomes, one approach considered by Brazilian authorities and scientists is more intensive use of the soils of the interior of the northeast of the country, which are generally sandy, with low contents of organic matter and low water holding capacity and are frequently affected by severe droughts. In this work, biochars produced from waste biomasses were tested for the improvement of these soils. The highest BET (Brunauer-Emmett-Teller) specific surface areas were observed for all biochars. In the pH range studied, the water hyacinth plants (WH) sample showed the most negative zeta potentials, as well as the highest water holding capacity (WHC) values, while the zeta potentials of two quartzarenic neosol soils were consistent with their WHC values. The results suggested that despite the effect of porosity on water retention, the zeta potential could be associated with the presence of negative charges by which hydrated cationic counterions were absorbed and retained. The surface energy and its polar and dispersive components were associated with water retention, with sugar cane bagasse, orange peel, and water hyacinth biochars presenting higher SE values and larger polar components.

## Introduction

The rapid expansion of agricultural activities in Brazil and the need for new agricultural land has led to advances into fundamental preservation biomes such as the Amazon, the Atlantic Forest, and the Pantanal (marshland). The inclusion of the Brazilian Cerrado as an important agricultural region has greatly expanded production. However, the lack of frequent rainfall makes crop and livestock farming problematic in the interior of the Northeast region of Brazil. The problem is exacerbated by the fact that the soils of the region are typically sandy and low in organic matter, resulting in poor water holding capacity (WHC). With the aim of improving the WHC of soils in the Northeast, the Post-Graduate Programs in Chemistry of the Federal University of Sergipe (Northeast Brazil) and the Federal University of Paraná (South Brazil) implemented a pilot project using biochar as a soil modifier.

In a book chapter published by the American Chemical Society^1^, it was observed that the addition of biochar, obtained cheaply from agricultural waste biomasses and produced on the farms themselves, increased the WHCs of these soils.

The use of pyrolyzed biomass residues in soils arose from research studies of the Amazonian soil known as “Terra Preta de Índios” (TPI). These soils, which were used by a number of tribes and covered large areas, were enriched with biomass charred by farmers. Some of these black and fertile patches are thought to be around 7000 years old and contain three times more nitrogen (N) and phosphorus (P) than the surrounding soils, and eighteen times more organic matter^2^. In 2009, we coordinated an international group of scientists to show how the lessons from the TPI of the Amazon region could help to improve tropical agriculture in Brazil^3^. Following investigation of the TPI, research studies have been directed towards the production of a material that imitates the TPI, denoted biochar^4^.

Biochar is produced by the thermal decomposition of biomass in an oxygen-deficient atmosphere over a wide range of temperatures, from 300 to 1000 °C^5^, in a process known as pyrolysis. It is a fine-grained carbonaceous material with high organic carbon content and is largely resistant to chemical and biological decomposition (mineralization)^6^. It can be produced from materials of animal origin, such as manure, as well as from waste from the paper and cellulose industry, agricultural residues such as coconut shells and sugarcane bagasse, and solid organic wastes such as sewage sludge, among others^1,7^.

Biochar has been used as a soil corrector and can influence soil properties and processes^6^. Several studies have shown that the presence of biochar in the soil can increase the availability of nutrients^8^, microbial activity, water retention^9–11^, and carbon sequestration^12^, while it may reduce fertilizer requirements, greenhouse gas emissions^13,14^, nutrient leaching, and erosion^15,16^. The use of biochar in agricultural soils has recently been suggested as an effective long-term tool to reduce the negative impacts of drought in the Brazilian Northeast, improving soil WHC^1^.

The addition of biochar to the soil can have direct and indirect effects on the retention of water in the soil, which can be of short or long duration. The direct effect is related to its large internal surface area and the high amount of residual pores, where water is retained by capillarity. This improves overall soil porosity and increases the soil water content, decreasing the mobility of the water and reducing water stress in plants. An indirect effect is improvement of soil aggregation and structure, consequently affecting the water retention capacity of the soil^17,18^.

Given that its behavior in the soil is highly complex, it is necessary to characterize biochars obtained from different feedstocks at relatively low temperatures (350 °C) in terms of their surface properties and residual pores, establishing relations between such properties and changes in fertility and WHC of semiarid soils such as those from the Brazilian Northeast region. The aim of the present work was to investigate the WHC of different biochars, identifying the main factors affecting this property.

## Results and Discussion

In this section, we will first characterize the porosity and surface area of different samples of biochar. We will then discuss the cation exchange capacity and zeta potential. Finally, the apparent free energy of the biochar surface will be determined. All these results will be correlated in order to identify the significant factors affecting the WHC of the material.

**Figure 1:**
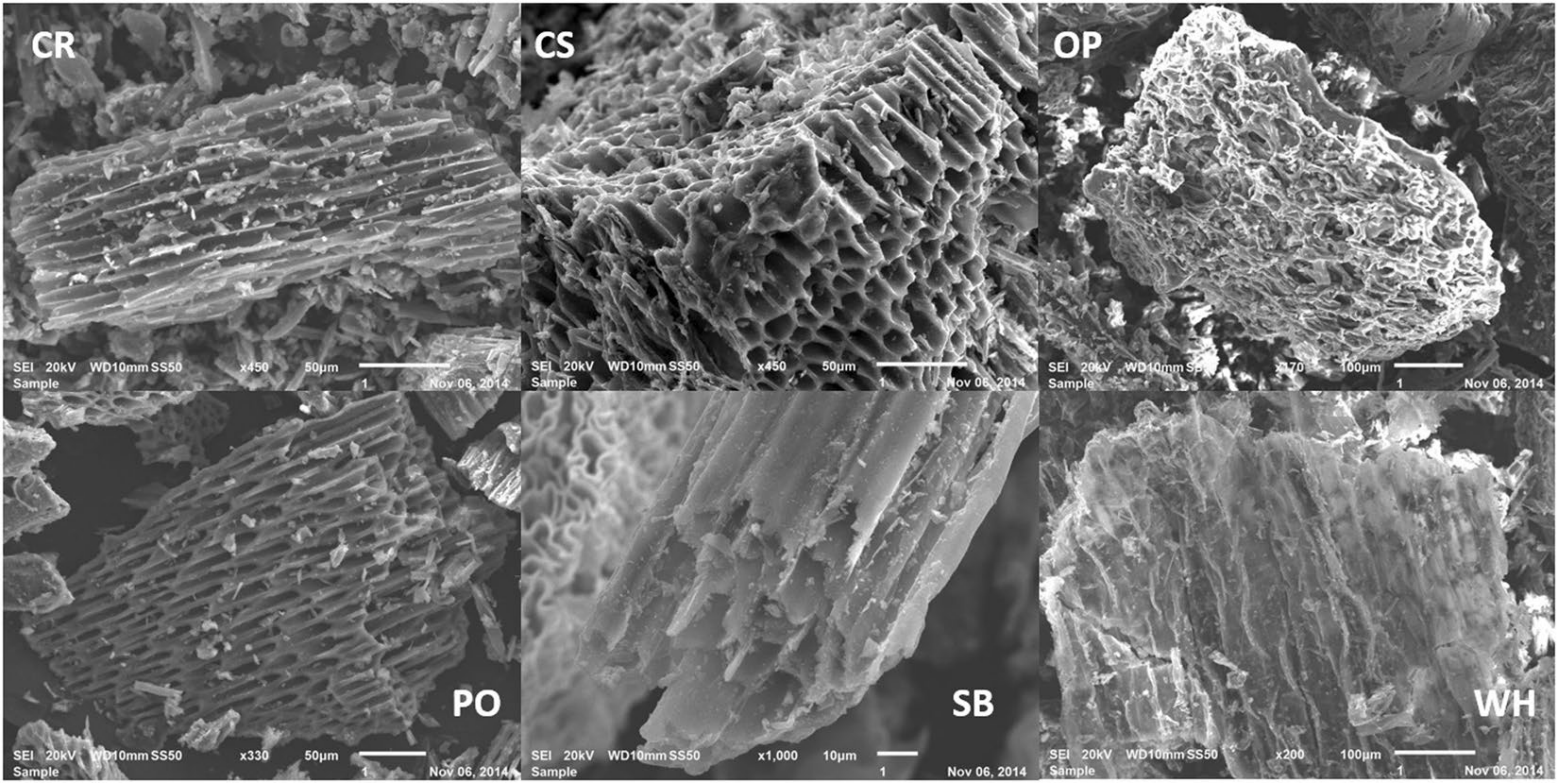
Scanning electron micrographs of biochar samples at magnifications of 450× (CR, CS), 170× (OP), 330× (PO), 1000× (SB), and 200× (WH). (CR = charcoal fines; CS = coconut shell; OP = orange peel; PO = palm oil bunch; SB = sugarcane bagasse; WH = water hyacinth).

Figure 1 shows scanning electron microscopy (SEM) images of the biochar samples at different magnifications. The slow pyrolysis, which was a focus of this study, caused the release of volatile organic matter, hemicellulose, and lignin, together with shrinkage, melting, and cracking, which improved the porosity of the materials^19,20^. The surface morphologies of all the biochars were highly heterogeneous and structurally complex, with many pores of different diameters. According to Dehkhoda *et al*.^21^, biochar presents an extremely complex network of pores and channels, together with a fibrous surface.

The biochar samples presented different pore sizes, which were approximately 10 μm for the charcoal fines (CR) and coconut shells (CS) samples, and approximately 6 μm for the oil palm bunch (PO) and sugarcane bagasse (SB) samples. Only the water hyacinth plants (WH) sample presented a rough surface, probably due to collapse of the pores and filling of the porous system with ash. The type of biomass and the pyrolysis conditions used influence the surface morphology and the physical properties of biochar^22^. In all cases, the majority of the pores can be classified as storage pores (0.5 to 50 μm) capable of holding water available to plants, with the water usually occupying pores with diameters between 0.5 and 50 μm and improving the retention of nutrients in the soil^23^.

The results of the BET (Brunauer-Emmett-Teller method) specific surface area (SSA) analyses are provided in Table 1. The biochars produced by slow pyrolysis at 350 °C showed an average porosity ≤10 μm (Fig. 1). The SSA and total pore volume (TPV) values obtained for the biochars were relatively low, varying from 43 to 186 m^2^ g^−1^ and from 9 × 10^−8^ to 1 × 10^−7^ m^3^ g^−1^, respectively. Charcoal fine (CR) SSA was different of the biochars (p < 0.1), but no differences were observed among the biochar samples. Related to the TPV there no differences between the samples (p < 0.1). The SSA results were in agreement with the scanning electron microscopy (Fig. 1) and the results published by Mangrich *et al*.^1^ that a high surface area of the biochar resulted in greater water holding capacity of these materials in the soil. Ronsse *et al*.^24^ evaluated biochars produced by the slow pyrolysis of different biomasses under a range of conditions. It was observed that at lower temperatures (300 and 450 °C), the specific surface area was generally low, but that it gradually increased in biochars produced using longer residence times. The relatively low surface areas observed for all samples were probably due to the inorganic material that partially filled or blocked the residual pores, as can be seen in the SEM images (Fig. 1). This was also observed by Rafiq *et al*.^25^, who investigated the influence of the pyrolysis temperature on the characteristics of biochar produced from maize hay. It was found that the surface area could increase temporarily when the biochar was added to soil, because the pore water leached minerals with low affinity to the biochar surface. Therefore, humic substances and other organic matter, heavy metals, and microorganisms may be expected to occupy the volumes of unoccupied pores.

**Table 1:** Specific surface area (SSA), total pore volume (TPV), and cation exchange capacity (CEC) of samples of biochar from different sources.

Sample^Ŧ^	SSA (m^2^ g^−1^)	TPV (m^3^ g^−1^)	CEC (cmol kg^−1^)
CR	43 ± 3^*^	4.1 × 10^−8^ ± 3.5 × 10^−8^	2 ± 0^**^
CS	157 ± 108	1.4 × 10^−7^ ± 8.5 × 10^−8^	13 ± 1^**^
OP	186 ± 89	9.4 × 10^−8^ ± 1.6 × 10^−8^	28 ± 1^**^
PO	173 ± 124	8.7 × 10^−8^ ± 1.4 × 10^−8^	35 ± 3^**#^
SB	159 ± 69	8.7 × 10^−8^ ± 2.3 × 10^−8^	7 ± 1^**^
WH	182 ± 103	5.5 × 10^−8^ ± 4.3 × 10^−8^	37 ± 2^**#^

^Ŧ^CR = charcoal fines; CS = coconut shell; OP = orange peel; PO = palm oil bunch; SB = sugarcane bagasse; WH = water hyacinth. Anova post-hoc Tukey test (^*^p < 0.1 or ^**^p < 0.01)^. #^samples PO and WH are not different between them (p > 0.01).

The elemental compositions and the atomic ratios of the biochars are shown in Table 2. The carbon content ranged from 45% to 60%, with the highest value for the PO sample. The low H/C ratios could be due to dehydration processes and decarboxylation associated with increased aromaticity and degree of condensation of the material^26,27^.

**Table 2:** Elemental analysis (C, H, N, O, and atomic ratios H/C, N/C, and O/C) of the biochar samples.

Sample^Ŧ^	C, H, N and atomic ratios H/C, N/C and O/C						
	%C	%H	%N	%O	H/C	N/C	O/C
CR	52 ± 0	3 ± 0	2 ± 0	43 ± 0	1	0	1
CS	59 ± 0	5 ± 0	1 ± 0	35 ± 0	1	0	0
OP	58 ± 1	5 ± 0	2 ± 0	35 ± 1	1	0	0
PO	60 ± 1	5 ± 0	1 ± 0	35 ± 1	1	0	0
SB	59 ± 1	4 ± 0	1 ± 0	37 ± 1	1	0	0
WH	45 ± 1	5 ± 0	4 ± 1	47 ± 0	1	0	1

^Ŧ^CR = charcoal fines; CS = coconut shell; OP = orange peel; PO = palm oil bunch; SB = sugarcane bagasse; WH = water hyacinth.

The cation exchange capacities (CECs) of biochar are different among the samples (p < 0.01). The biochar from palm oil bunch (PO) and water hyacinth (WH) presented higher CEC than the other samples, however, with no differences between the two samples. The CEC values showed high correlation with the O/C ratios of the biochar samples, with a higher O/C ratio being associated with a higher CEC value. A higher O/C ratio may indicate the presence of more hydroxyl, carboxylate, and carbonyl groups, which could contribute to higher CEC of the biochar. The CEC values for WH, PO, and OP were substantially higher than for the other biochar samples. These materials could be used to improve soil properties such as CEC, while at the same time contributing to the sequestration of carbon in the soil^28^. In general, lower hydrogen and oxygen contents were associated with greater hydrophobicity of the biochar, notably in the case of CR, in agreement with the formation of more aromatic compounds, as evidenced by the ^13^C NMR analyses of these biochars presented by Mangrich *et al*.^1^.

Oxidation of the biochar surface or a lower pyrolysis temperature can result in functional groups attached to the surface, composed primarily of oxygen, hydrogen, and carbon. These functional groups can bind with nutrients and minerals, while the fused carbon rings support redox reactions and shuttle electrons around the microbial community attached to the biochar surface, potentially enhancing microbial metabolism and the cycling of nutrients in soils. Most of the biochar samples showed pKa values typical of carboxylic acids and phenols, ranging from 3.74 to 11.40. These pKa values can be tentatively ascribed to various weak acidic groups that form hydrogen bonds^29^. The pKa values and the amounts of organic groups present in the biochar samples are listed in Table 3.

**Table 3:** Analyses of pKa and the amounts of organic groups present in the biochar samples, measured at 25 °C in 0.10 mol L^−1^ KCl. The values in parentheses are the variations of the measurements (last decimal place).

Sample^*^	pKa	Organic groups mmol g^−1^)	
		Partial	Total
CR	10.90 (4)	0.700 (3)	1.65
	8.70 (1)	0.320 (1)	
	6.20 (1)	0.270 (2)	
	4.16 (14)	0.362 (6)	
CS	10.61 (20)	0.204 (4)	0.20
OP	11.40 (4)	0.460 (7)	0.62
	9.45 (20)	0.160 (30)	
PO	10.93 (5)	0.978	0.98
SB	11.06 (6)	0.051 (7)	0.09
	9.23 (3)	0.024 (1)	
	6.70 (2)	0.007 (5)	
	3.74 (3)	0.011 (1)	
WH	10.90 (10)	0.780 (2)	1.19
	6.32 (1)	0.406 (22)	

^*^CR = charcoal fines; CS = coconut shell; OP = orange peel; PO = palm oil bunch; SB = sugarcane bagasse; WH = water hyacinth.

**Figure 2:**
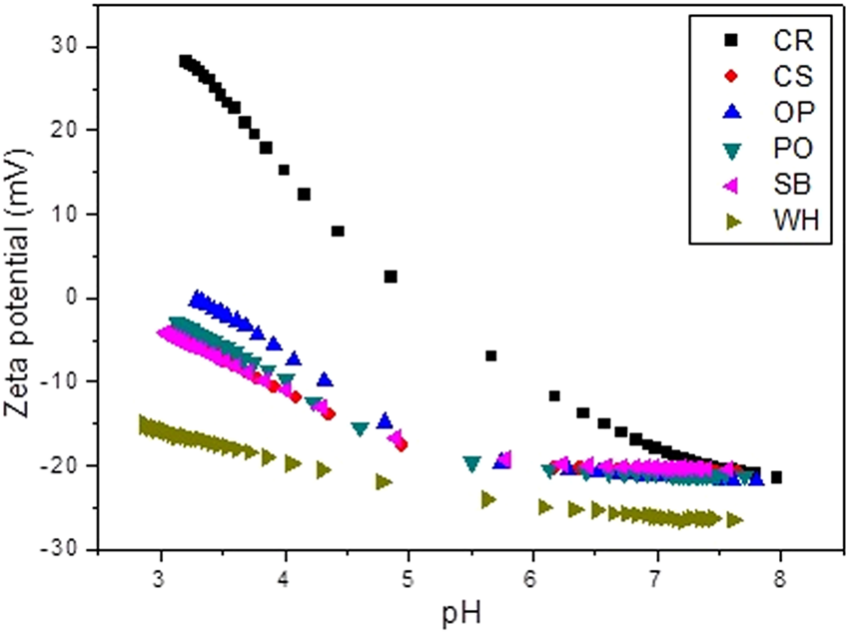
Zeta potentials of the biochar samples: CR = charcoal fines; CS = coconut shell; OP = orange peel; PO = palm oil bunch; SB = sugarcane bagasse; WH = water hyacinth.

**Figure 3:**
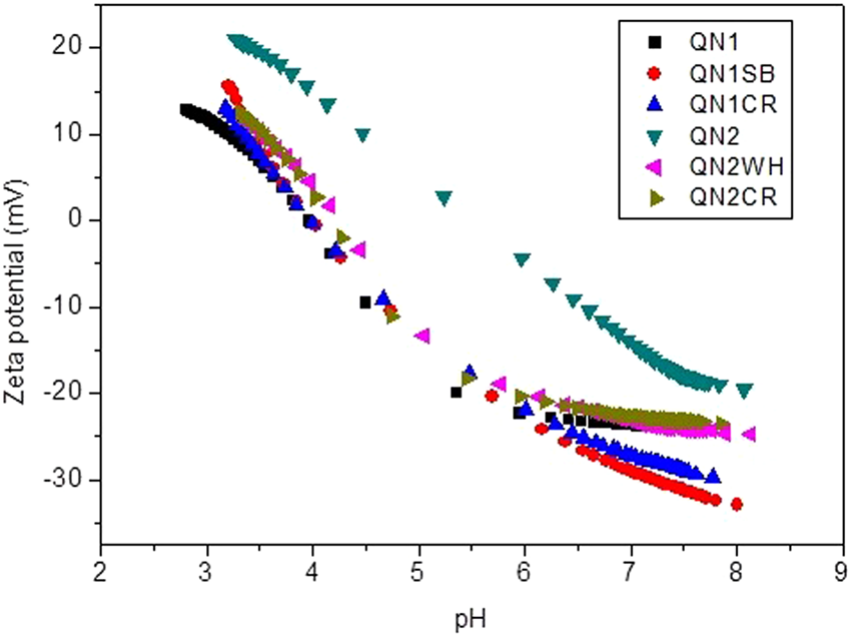
Zeta potentials of the soils and mixtures (soil + biochar): QN1 = quartzarenic neosol soil 1; QN1SB = quartzarenic neosol soil 1+ sugarcane bagasse biochar; QN1CR = quartzarenic neosol soil 1+ charcoal fines; QN2 = quartzarenic neosol soil 2; QN2WH = quartzarenic neosol soil 2+ water hyacinth biochar; QN2CR = quartzarenic neosol soil 2+ charcoal fines.

Another possible explanation for the enhanced water retention capacity of soils following the addition of biochar, as observed by Mangrich *et al*.^1^, is the electric potential at the hydrodynamic shear plane (zeta potential, ζ). Plots of ζ as a function of pH for the biochar samples are shown in Fig. 2. As expected, the ζ value decreased when the pH of the medium increased, due to the adsorption of OH^−^, Cl^−^, or other anions at the biochar surfaces^30^. However, these negative charges decreased in an acid medium, which could be attributed to reduced ionization of the weak acid groups present on the colloid surfaces, or the protonation of amine groups. All the biochar samples showed negative zeta potentials across a wide range of pH values, indicating the presence of negative surface charges^20^. At the pH values tested, sample WH (Fig. 2) showed the most negative zeta potentials, as well as the highest water holding capacity (WHC) following its addition to soils (as reported by Mangrich *et al*.^1^). The zeta potential values obtained for the other biochar samples were not significantly different. In Fig. 3, the same type of behaviour can be seen for soil QN1, which showed a lower zeta potential than soil QN2, in agreement with the WHC results presented by Mangrich *et al*.^1^.

The higher WHC of soil QN1 was due to the greater amounts of clay and carbon in its composition (Mangrich *et al*.^1^). These results suggest that despite the effect of porosity on water retention, the zeta potential could be associated with the presence of negative charges by which the hydrated cationic counterions were adsorbed and retained on the surfaces of the biochar or soil. This process should be helpful for improvement of the fertility of soils modified with biochars, indicating favourable surface characteristics for agricultural applications^31^.

Biochars CR and OP presented isoelectric points (IEP) of 4.7 and 3.3, respectively, while a value of 4.0 was obtained for all the other biochars and the mixtures with soil. The control soil samples (without biochar) had IEP values of 4 (soil QN1) and 5.1 (soil QN2). When the biochars were added to the soil, the organic acids of the biochar chemical structures were adsorbed by the mineral colloids in suspension, derived from the soils, causing an increase of negative charges and lowering the IEP.

The OP sample showed the highest IEP, probably due to the low amount of oxygen-containing functional groups on the surface, as evidenced by the elemental analysis. In addition, the IEP of the other biochar samples was not reached in the experimental pH window used, suggesting values <3. This could be attributed to the higher content of oxygen-containing surface functional groups in the case of the WH sample, compared to the other samples^32,33^.

The contact angle values for the biochars and soil samples in two different polarity solvents are shown in Table 4. For each liquid, the polar and dispersive components were calculated from the liquid-solid contact angles, in order to determine the free energy of the solid surface. In the case of a droplet of purified water (Milli-Q system, Millipore), at the interface the polar component is of major importance and can be subject to capillary forces, depending on the hydrophobicity/hydrophilicity ratio of the biochar surface.

**Table 4:** Contact angles (θ) formed by drops of H_2_O and diiodomethane, and polar and dispersive components of the surface energy for the different biochar and soil samples.

Sample^*^	θ (°)		^#^OWRK method		
	H_2_O	Diiodomethane	SE (mJ m^−2^)	Dispersive (mJ m^−2^)	Polar (mJ m^−2^)
CR	110 ± 9	9 ± 2	59	57	2
CS	114 ± 1	10 ± 2	61	58	3
OP	131 ± 2	19 ± 3	69	60	9
PO	91 ± 9	19 ± 3	49	49	0
SB	132 ± 7	18 ± 4	71	61	10
WH	124 ± 13	19 ± 2	64	58	6
QN1	57 ± 5	17 ± 2	54	40	15
QN2	59 ± 4	12 ± 3	54	41	13

^*^CR = charcoal fines; CS = coconut shell; OP = orange peel; PO = palm oil bunch; SB = sugarcane bagasse; WH = water hyacinth; QN1 = quartzarenic neosol soil 1; QN2 = quartzarenic neosol soil 2. ^#^Owens-Wendt-Rabel-Kaelble theory.

Soil samples quartzarenic neosol soil 1(QN1) and quartzarenic neosol soil 2(QN2) were wetted with water, forming contact angles lower than 90°. None of the biochar surfaces showed good interaction with the polar liquid (water), forming contact angles higher than 90°. This clearly demonstrated that the work of adhesion ($Wa={\gamma }_{s}\,+\,{\gamma }_{l}-\,{\gamma }_{sl}={\gamma }_{l}\,(1+cos{\rm{\theta }}$)) between the liquid and the surface was much lower than the work of cohesion in the liquid ($Wc=2{\gamma }_{l}$). The sugarcane bagasse sample (SB) was more hydrophobic than the other samples, since it had the highest water contact angle, indicating a less wettable surface. The opposite occurred using a highly dispersive (nonpolar) liquid (diiodomethane), with samples CR (charcoal fines) and CS (coconut shell) showing the lowest contact angles of 10° and 9°, respectively (Table 4).

Calculation of the surface energies of the biochars required measurement of the contact angle between the liquid and the solid^34,35^. There are several theories to describe the surface energy of a solid. The Owens-Wendt-Rabel-Kaelble (OWRK) theory describes the polar and dispersive components of the surface energy, which were calculated from the contact angles obtained for the different materials (Table 4). All the samples showed high dispersive component values, varying between 40 and 61 mJ m^−2^. The values of the polar components for these materials were lower for the biochars (ranging from 0 to 10 mJ m^−2^), compared to the soils (15 mJ m^−2^ for QN1 (Quartzarenic neosol soil 1) and 13 mJ m^−2^ for QN2 (Quartzarenic neosol soil 2)). The sample with the highest surface energy (SE) and polar component was SB (sugarcane bagasse), followed by OP (orange peel) and WH (water hyacinth plants).

Based on all the results presented above, it was not possible to attribute the water holding capacities of the biochars to any one parameter. The porosity and surface area were apparently first order factors that showed direct correlation with water retention in the soil, with the highest values for samples OP = orange peel, WH = water hyacinth plants, and PO = palm oil bunch. However, secondary aspects should also be considered. The zeta potential was a significant parameter, especially for the WH sample, while the values were not significantly different among the other samples. The WH = water hyacinth plants biochar also presented the highest specific surface area (SSA) and cation exchange capacity (CEC) values, suggesting effects that were synergistic with porosity and surface area. The solid surface energy and its polar and dispersive components could also be associated with water retention, since the SB = sugarcane bagasse, OP = orange peel, and WH = water hyacinth plants samples presented higher surface energy (SE) values and larger polar components, compared to the other biochar samples. According to Mangrich *et al*.^1^, these were the biochars with the highest water holding capacity (WHCs).

## Conclusions

Two main groups of factors were associated with the water holding capacities of the biochars. The porosity, characterized using microscopy and BET isotherms, is well known as an important factor affecting water retention. However, other secondary parameters also influenced the WHCs of the biochars, including the zeta potential and the cation exchange capacity. These two important factors were associated with the adsorption of hydrated ions on the biochar surface and were correlated with the water retention. All the biochars were mostly hydrophobic, with only a small polar component. This component also appeared to contribute to the water holding capacity, since the samples with larger polar components showed greater water retention efficiencies.

## Methods

### Preparation of the biochars

The biomasses used were green coconut shells (*Cocos nucifera* - CS), orange peel (*Citrus sinensis* - OP), oil palm bunch (*Elaeis guineensis* - PO), sugarcane bagasse (*Saccharum officinarum* - SB), water hyacinth plants (*Eichhornia crassipes* - WH), and charcoal fines (CR). These materials were dried in an oven (Model G3, Gehaka, Brazil) at 105 °C for 24 h. The biochars were then prepared by pyrolysis of the materials at low temperature (350 °C) under a controlled atmosphere. For this, the samples were placed in a tunnel oven (Model FT-40, EDG, Brazil) and the system was completely sealed, except for an outlet for bubbling of the gases into distilled water. Only the oxygen initially contained in the pyrolysis tube was available during the thermochemical reaction. The heating rate used was 5 °C min^−1^. The total pyrolysis time was approximately 1 h. After preparation, the biochars were reduced to a particle size of 2 mm using a knife mill (Willye Star FT 50, Fortinox, Brazil).

### Scanning electron microscopy (SEM)

The biochar samples were first metallized and were then analyzed using a JSM 6360 LV scanning electron microscope (JEOL, Japan) operating at 15 kV. The materials were metallized using a Balzers Union SCD 030 system, at the Mineralogy Laboratory (LAMIR) of UFPR. The SEM images were acquired using the proprietary JEOL software.

### Specific surface area, pore volume, and average pore size

Nitrogen adsorption at 77 K was used to determine the biochar specific surface area, pore volume, and average pore size, using the BET (Brunauer-Emmett-Teller) method. The analyses were performed using a NOVA 1200 instrument (Quantachrome, USA), at the Chemistry Department of the Federal University of Sergipe (DQ/UFS). This method is based on determination of the volumes of gas adsorbed and desorbed at different relative pressures. Before analysis, all the biochar and charcoal fines samples were submitted to degasification at 150 °C for 2 h.

### Elemental analysis

Quantification of carbon, hydrogen, and nitrogen was performed simultaneously using an elemental analyzer (Model PE2400 CHNS/O, PerkinElmer, USA), with around 2–2.5 mg of each sample. A calibration curve was obtained using an acetonitrile standard. The combustion time was 600 s, at a temperature of 925 °C, under a flow of helium gas. All the analyses were performed in duplicate. The oxygen (O) content was inferred by difference (Equation 1).

1 $$( \% O)=100-[( \% C)+( \% N)+( \% H)]$$

### Cation exchange capacity (CEC)

The cation exchange capacity (CEC) describes the total amount of exchangeable cations that could be retained by the biochars or the charcoal fines. The CEC was calculated in terms of milliequivalents per 100 g of dried biochar or charcoal fines. For this analysis, a 2 g portion of sample was mixed with 100 mL of 0.5 mol L^−1^ HCl in a 500 mL Erlenmeyer flask, in order to remove weakly bound metal cations such as Na^+^ and K^+^, known in agronomy as bases, and to saturate the sorption sites of the material with H^+^. The vial was sealed and mechanically shaken at 150 rpm for 30 min at 25 °C in an incubator (Model TE-421, Tecnal, USA). The excess acidic aqueous solution was then removed by vacuum filtration using plain filter paper and the material was washed with 100 mL portions of water containing a few drops of 1% (m/v) AgNO_3_ to prevent precipitation. The sample was again transferred to the 500 mL Erlenmeyer flask and the adsorbed H^+^ ions were replaced with Ba^2+^ by the addition of 100 mL of 0.35 mol L^−1^ barium acetate, with stirring for 15 min. The material was filtered and washed with three 100 mL portions of water. The solid was discarded and the filtrate was titrated against 0.1 mol L^−1^ NaOH solution, using 5 drops of phenolphthalein as indicator^27^. The CEC was calculated using Equation 2.

2 $$CEC=\frac{V(mL)x\,0.1\,mol.{L}^{-1}(NaOH)x\,100}{2g}$$

### Potentiometric titrations

Quantitative measurements of pK_a_ and the amounts (in mmol g^−1^) of organic groups present in the biochar and charcoal fines samples were performed by potentiometric titrations using an automatic titrator (Titrino Plus 350, Metrohm, Switzerland) equipped with an Ag/AgCl combination electrode. The instrument was calibrated using a dilute solution of HCl (0.010 mol L^−1^, μ = 0.100), in order to directly read –log[H^+^]. A thermostatic bath (Model MQBTC99-20, Microquímica, Brazil) was used to control the cell temperature at 25 °C. Samples of 0.100 g of the biochars or charcoal fines were added to the cell, together with 20.0 mL of bi-distilled and boiled water, 20.0 mL of HCl (0.100 mol L^−1^), and 0.298 g of KCl (to control the ionic strength). This suspension was titrated up to pH 11 with a 0.100 mol L^−1^ CO_2_-free KOH standard solution. Computations of data from triplicate experiments were performed using the BEST7 program^36^. This FORTRAN program is used for the determination of equilibrium constants and quantification of the percentage of each species, based on the titration curves. It minimizes the standard deviation of the fit between the observed and calculated pH values for the entire titration curve. The technique has been used to determine acidic and basic groups present in natural samples such as humic acid, fulvic acid, and others^29,37^.

### Zeta potential measurements

The zeta potentials of the biochars, charcoal fines, and blended samples were determined using a Stabino particle charge titration analyzer (Microtrac, Germany). The samples were dried in an oven (Model G3, Gehaka, Brazil) at 105 °C for 24 h. A suspension of 5 mg of sample was prepared in 50 mL of 0.01 mol L^−1^ KCl. Solutions of NaOH and HCl (0.01 mol L^−1^) were used to vary the pH from 3 to 8. Every 300 s (for the biochars and the charcoal fines) and 200 s (for the blends), 40 μL of a NaOH solution was added in order to raise the pH to 8, and HCl solution was added in order to lower the pH to 3. This procedure enabled a zeta potential plot to be obtained as a function of pH. The isoelectric potential (the electric potential equal to zero at the hydrodynamic shear plane) of the sample was then determined from this graph.

### Contact angle measurements

An OCA Contact Angle System precision tensiometer (DataPhysics, Germany) was used to determine the contact angle of the droplets on the surfaces of the biochar, charcoal fines, and soil samples, and the contact angle was also obtained by image analysis. The methodology used was based on that described by Bachmann *et al*.^38^. The angles on the right and left sides of the droplet images for the different solutions were calculated and automatically averaged, providing the equilibrium contact angle after reaching a constant value for at least two minutes. The hydrophobicities of the materials were evaluated using different polarity solvents: water and diiodomethane. The volumes of the droplets applied to the surfaces of the materials were 10 μL (water) and 6 μL (diiodomethane), delivered using a 50 μL graduated syringe. For these evaluations, glass slides were thoroughly washed and dried, so that no particles remained that could compromise the evaluations. Each slide was wrapped with double-sided adhesive tape, which was then covered with a layer of soil or biochar particles. In preliminary experiments, it was found that the tape provided contact angles of approximately 90° against water, and did not affect the contact angles obtained with the sample particles attached to the surface. The system used for the evaluations consisted of a digital camera fixed in front of a platform, where the slides were positioned in order to be able to view the contact angle of the droplets delivered from the 50 μL syringe suspended above and very close to the slide. A single filament lamp was positioned in front of the slide holder in order to ensure a sharp image. Image acquisition was controlled by SCA 20 software (DataPhysics, Germany). For each treatment, five droplets were used, resulting in 10 angle measurements. Young’s equation was used to relate the contact angle (θ) to the surface tension of the liquid (${\gamma }_{l}$), the interfacial tension (${\gamma }_{sl}$) between the liquid and the solid, and the surface free energy (${\gamma }_{s}$) of the solid (Equation 3).

3 $${\gamma }_{s}=\,{\gamma }_{sl}+\,{\gamma }_{l}cos{\rm{\theta }}$$

As it is impossible to obtain ${\gamma }_{s}$ directly from Equation 3, it is necessary to determine ${\gamma }_{sl}$ based on ${\gamma }_{s}$ and ${\gamma }_{l}$, using the geometric mean of the disperse component, ${\gamma }^{D}$, and the polar component, ${\gamma }^{P}$, of ${\gamma }_{l}$ or ${\gamma }_{s}$ (Equation 4).

4 $${\gamma }_{sl}=\,{\gamma }_{s}+\,{\gamma }_{l}-2(\sqrt{{\gamma }_{s}^{D}.{\gamma }_{l}^{D}}+\,\sqrt{{\gamma }_{s}^{P}.{\gamma }_{l}^{P}})$$

At least two liquids with known disperse and polar components of the surface tension are required in order to determine the surface free energy of the solid, and at least one of the liquids must have a polar component higher than zero^39,40^.

### Statistical analysis

The data is presented as the mean of multiple independent experiments (±SD). Significance was determined using the one-way ANOVA test with post-hoc Tukey’s multiple comparison test. *p values lower than 0.1 or **p values lower than 0.01 were considered statistically significant and are indicated in the tables.
